# History of the Last Illness of M. Broussais

**Published:** 1840-01

**Authors:** 


### *History of the last illness of M. Broussais.*

The editor of the Medico-Chirurgical Review, to whom we are indebted for this extract from the French Journal, very plausibly suggests that mischief was done in the case of M. Broussais, carcinomatous in its character, by the artificial dilatation of the gut by means of large bougies, and the application of caustic to its surface, and cautions the young surgeon against the employment of very energetic and hasty measures in the treatment of such diseases.

"It would seem that, after the death of the illustrious medical reformer, various reports were in circulation that his disease had been mismanaged, and that his health and constitution had suffered more from the medical means that had been employed, than from the severity of the malady itself. M. *Amussat*, his medical attendant, has therefore felt himself called upon to exculpate himself by laying before the medical public a detailed report of his distinguished patient's case, and of the practice which he had pursued in treating it. The case is an instructive one, and the abridged report which we propose to lay before our readers, may probably interest them on more than one account.

For many years M. *Broussais* had been subject occasionally to attacks of severe pain, situated apparently in the pyloric extremity of the stomach, and of diarrhœa recurring at intervals. His appetite and digestion however, were almost uniformly good.

During the last two years or so of his life, he had experienced a gradually-increasing difficulty in the evacuation of the bowels; and at the same time pains at the neck of the bladder, and some degree of dysentery were frequently experienced. In April, 1838, he first consulted his friend, M. *Amussat*, who ascertained by examination, that the anterior wall of the lower part of the rectum was hard, thickened, and knobby, and who, (he tells us himself,) augured unfavorably of the case from his very first visit, although he assured his friend that his disease was only hæmorrhoids, and would be easily cured by means of ligature.

In consequence, however, of M. *Broussais's* objections to any operation, the use only of bougies, for the purpose of dilating the gut was resorted to. Considerable relief was obtained from this practice. A few weeks subsequently, M. *Amussat*, believing that more active measures were necessary, requested that MM. *Breschet* and *Sanson* be called to consult with him upon the case. It was agreed that the same treatment should be continued as hitherto, and that, if the vegetations on the gut increased much in size, recourse should be had to the use of the ligature and of caustic.

In July, the largest one of the tumors or vegetations, which was constantly protruded with a good deal of distress at each evacuation, was tied: it quickly came away, and the patient was considerably relieved in consequence. A few days after this operation, another tumor in every respect like the preceding one, began to cause the same uueasiness; and M. *Amussat* therefore resolved to get rid of it also by ligature.

It would seem, however, that the state of the parts was at this time even more unfavorable than it had been; for it is mentioned in the report;—"the disease had attacked the entire circumference of the bowel, and had reached down to the sphincter: the mucous membrane was observed to be of a shining yellow color and to be much altered in its texture." A ligature was applied to the other tumor: but, as it could not be put high enough up so as to surround its base, M. *Amussat* attempted to destroy the pedicle by squeezing it between the blades of a pair of forceps.

This operation did not cause much uneasiness; but the examination of the gut afterwards was acutely painful. The bougies and tents were again had recouase to.

On the 13th of August, a small vegetation, which was constricted by the sphincter, was excised.

The report continues:—

"The inferior orifice of the rectum had been cleared from all obstructions, and was now readily dilatable. Not so, however, with the diseased portion of the gut. As already stated, the carcinomatous affection had attacked its whole circumference, and the diameter of the passage was so much diminished at the upper part, that the alvine evacuations could scarcely pass through. Enemata produced little or no benefit; and it was only by the use of the bougies and of purgative medicines that the bowels could be relieved."

As the vegetations appeared to be re-forming, M. *Amussat* began at this time to apply lunar-caustic to the inferior constricted part of the gut, and chiefly to its posterior wall, with the view, says he, *de detourner l'irritation qui se propageait a la vessie.* The urinary distress was speedily much relieved: Broussais himself has noted in his diary at this time, *Je n'ai plus de spasme dans la vessie; quand il y a de l'urine, je la rends.*

During the next four weeks, the free application of the caustic* to the diseased surface of the rectum was made four or five times: upon one or two occasions it caused very great pain.

During all this time, the patient seems to have been becoming weaker: his feet began to swell, and little or no progress had been made in widening the passage of the rectum. Indeed it is stated in the report of the case by M. *Amussat*, that on exploring the rectum after the eighth application of the caustic, he found the upper opening of the constricted part so much narrowed that the fæculent matter, collected above the point, could not pass through at all.

After this date, M. *Broussais* went to his country-house

*M. *Amussat* had a large *porte-caustique* made on purpose, as the extent of the diseased surface, which it was necessary to cauterise, was so considerable.

at *Vitry*, expecting that his general health might be improved by the change. He gradually, however, became weaker and weaker, and on the 16th of Nov., after an ineffectual attempt to relieve his bowels, he fell into an apoplectic stupor, and died in a few hours afterwards.

*Dissection.*—We shall only notice the state of the rectum. The extent of the diseased portion was for about four inches or so from the anus upwards. At one part the surface exhibited a state of pulpy *ramollissement*, resembling, somewhat the appearance of cerebriform degeneration.* The submucous cellular tissue was partially infiltrated with grumous purulent matter.

On the posterior part of the bowel, there was found a rupture of about an inch in extent; but this had taken place when the bowel was drawn away from its attachments.

At no part of the diseased portion was the gut observed to be much constricted: and, judging merely from the *post mortem* state of the parts, one could never have anticipated the degree of obstruction that had been present during life. The portion however of the rectum and of the sigmoid flexure of the colon immediately above or beyond the diseased parts, was found to be much more distended than it usually is.—*Gazette Medicale.*

*We must not omit to mention that the putrefaction of the body was already considerably advanced, when the examination took place.

